# Supplementary material for: Revealing the mechanism and function underlying pairwise temporal coupling in collective motion
Source: Nat Commun. 2024 May 22;15:4356. doi: 10.1038/s41467-024-48458-z (PMC11111445; doi:10.1038/s41467-024-48458-z)
Supplement: Supplementary file 1 — Supplementary Information [file 41467_2024_48458_MOESM1_ESM.pdf]

Supplementary Information

Revealing the mechanism and function underlying pairwise temporal coupling in collective motion

Guy Amichay, Liang Li, Máté Nagy and Iain D. Couzin

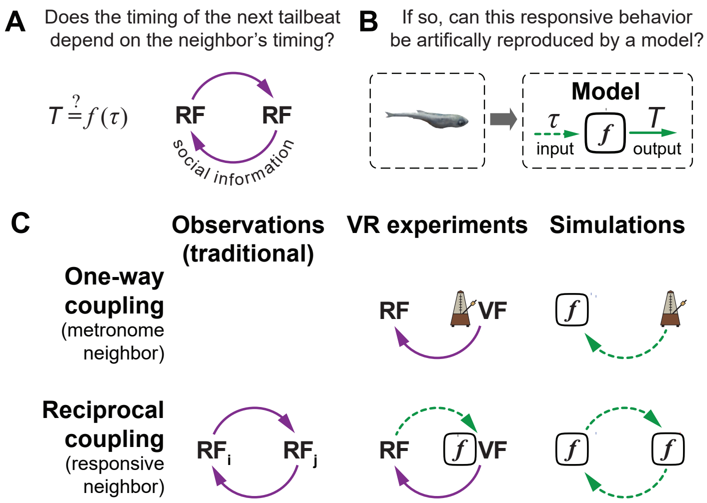

Supplementary Fig. 1: Explanation of the different coupling schemes in the study. (A-B) Schematic representation of the main questions in our system related to behavioral reciprocity (A) and the modeling approach (B). (C) The difference between one- and two-way coupling, in the different methods that we used (observations, controlled experiments (in VR), and modeling/simulation).

.

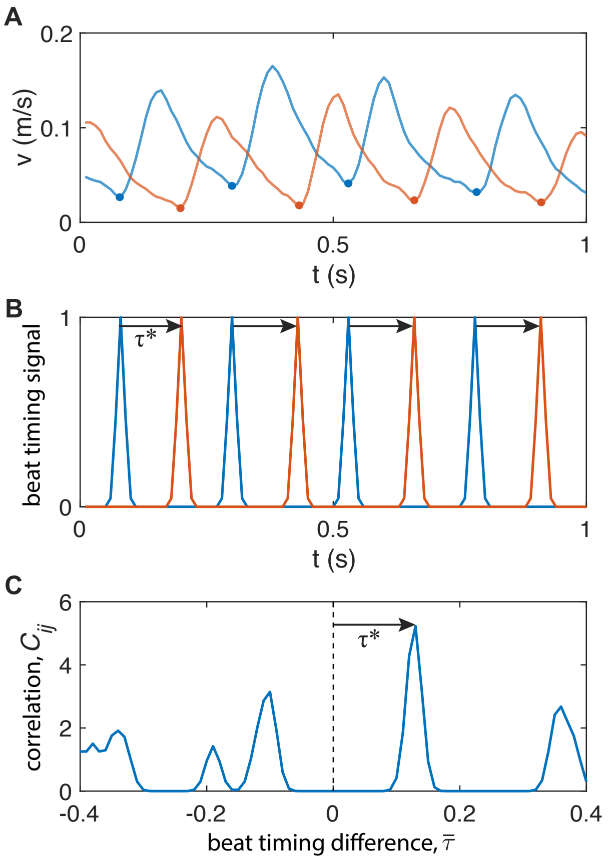

Supplementary Fig. 2: Explanation of the procedure for calculating the correlation function. (A) The speed of each fish over time. (B) The timing of minima of the speed in (A). We transform the speed profile into a binary signal of beat timings–with a value of 1 in the frames of minima. We pad each minimum by expanding it to a small Gaussian around the detected frame, to account for noise. (C) We correlate (calculate the dot product) of the signals in (B) with varying delays relative to each other τ, to obtain C_{ij}. The highest value of the correlation function defined the time delay, τ^* that is depicted as black arrows in (B) and (C).

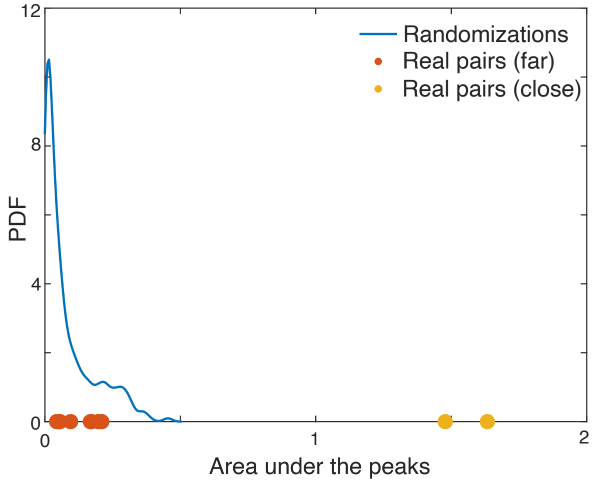

Supplementary Fig. 3: Statistical procedure used for assessing significance. Comparison of the peaks in the correlation functions. After shifting the correlation function by subtracting its mean ($C_{\text{shift}} = C - \langle C \rangle$), we calculate the integrals of the resultant peaks by $\int_{-0.3s}^{0.3s} C_{\text{shift}}$.

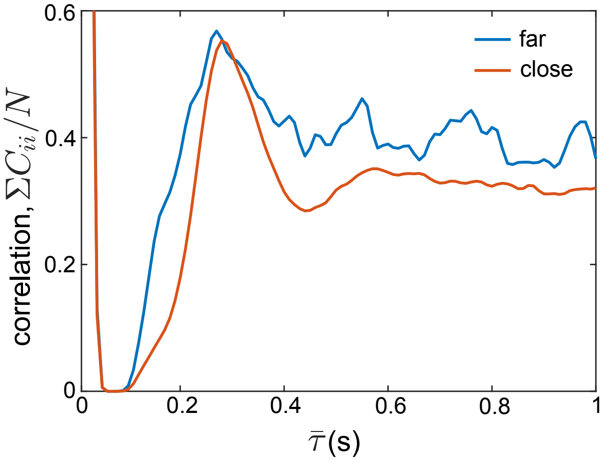

Supplementary Fig. 4: The autocorrelation of beat times when pairs of fish were close (d<4cm) and far away (d>10cm).

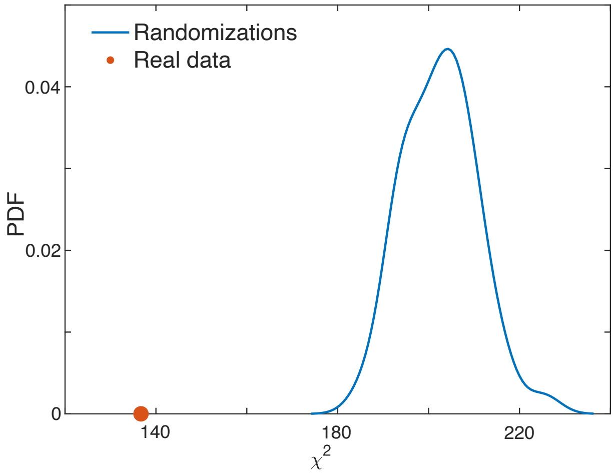

Supplementary Fig. 5: Statistical procedure used for assessing significance. Comparison of χ^2 values obtained for fitting y=2x on the PRC (denoted by a red dot) vs equivalent values obtained when shuffling the pairs (randomizing the data).

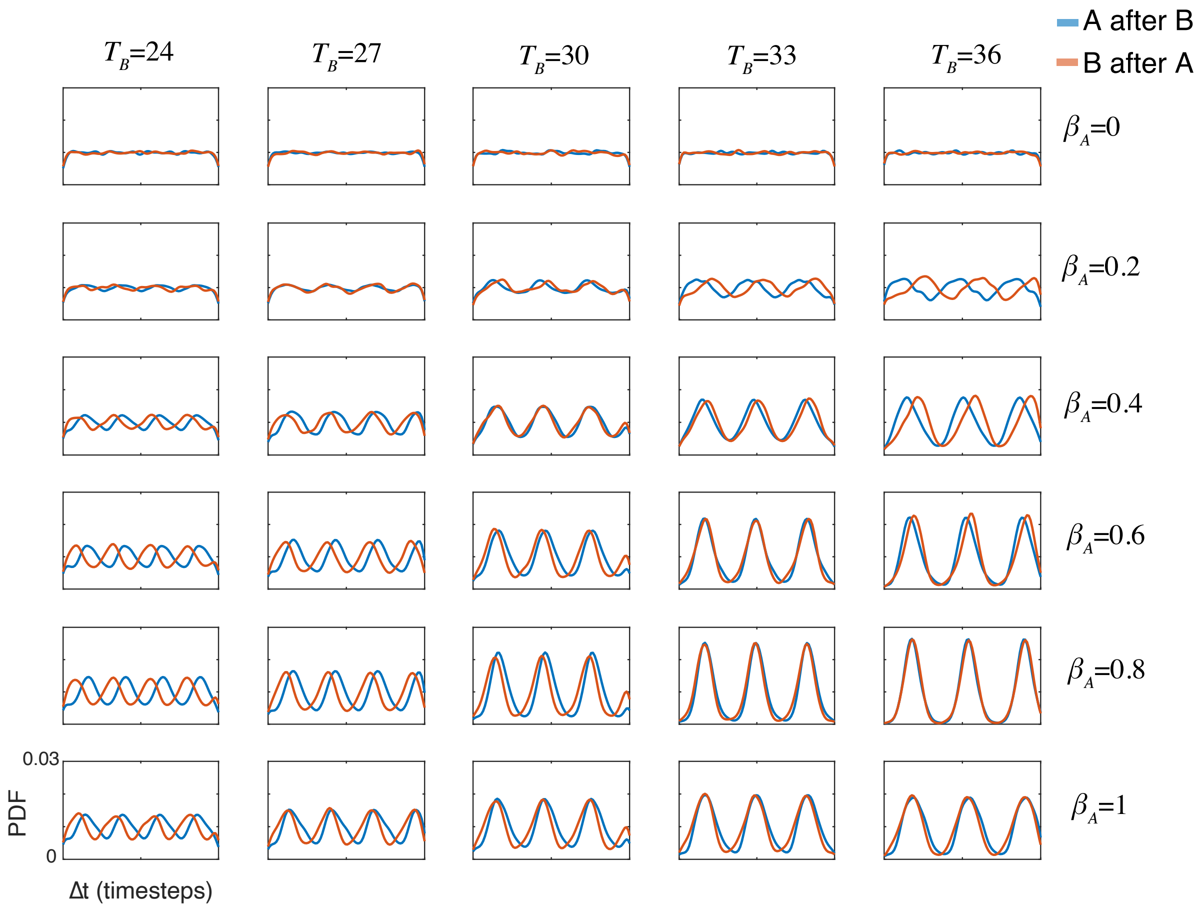

Supplementary Fig. 6: The distribution of time differences Δt between the two oscillators in the unidirectional model. Each plot corresponds to a different setting of β_A (the reciprocal oscillator) and T_B (the metronome) in timesteps.

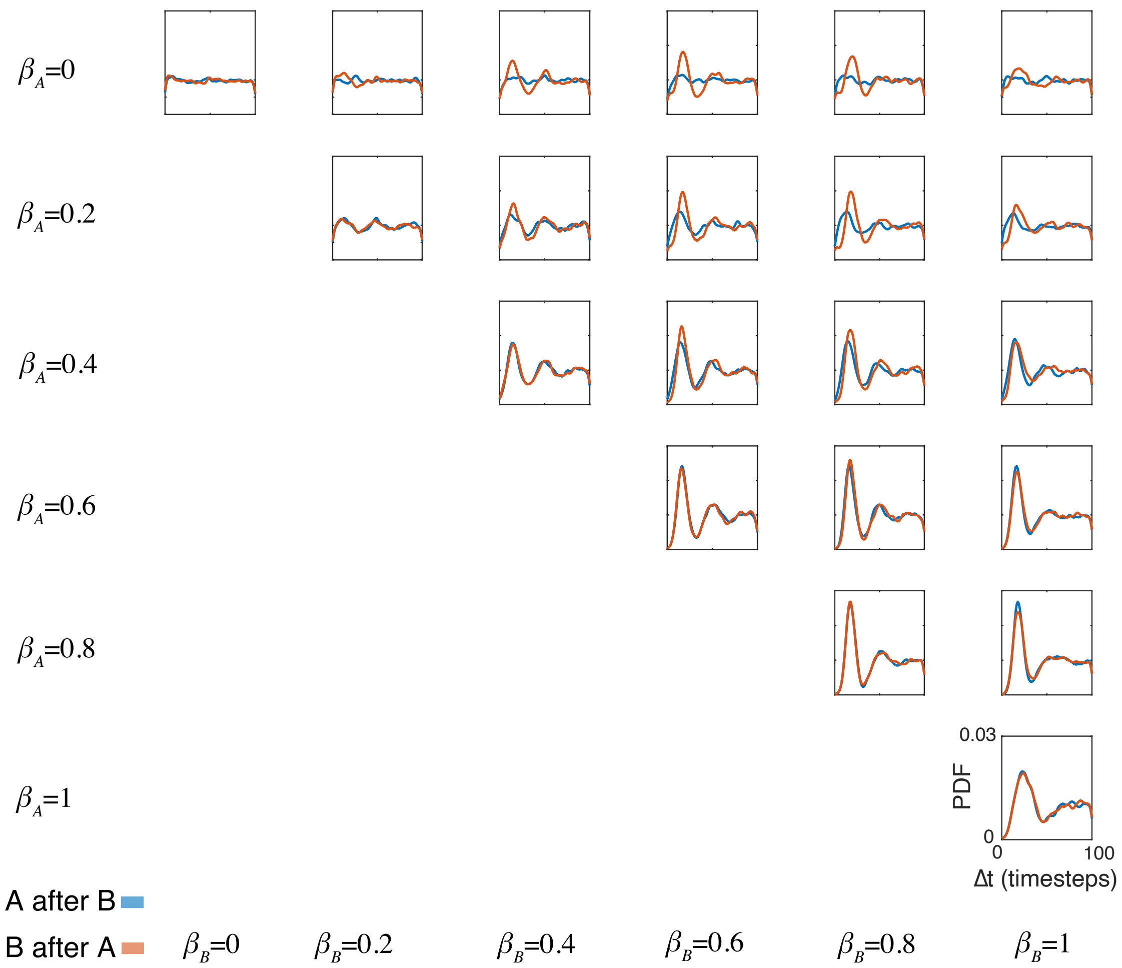

Supplementary Fig. 7: The distribution of time differences Δt between the two oscillators in the bidirectional model. Each plot corresponds to a different setting of β per each oscillator. Some plots are not shown due to symmetry.

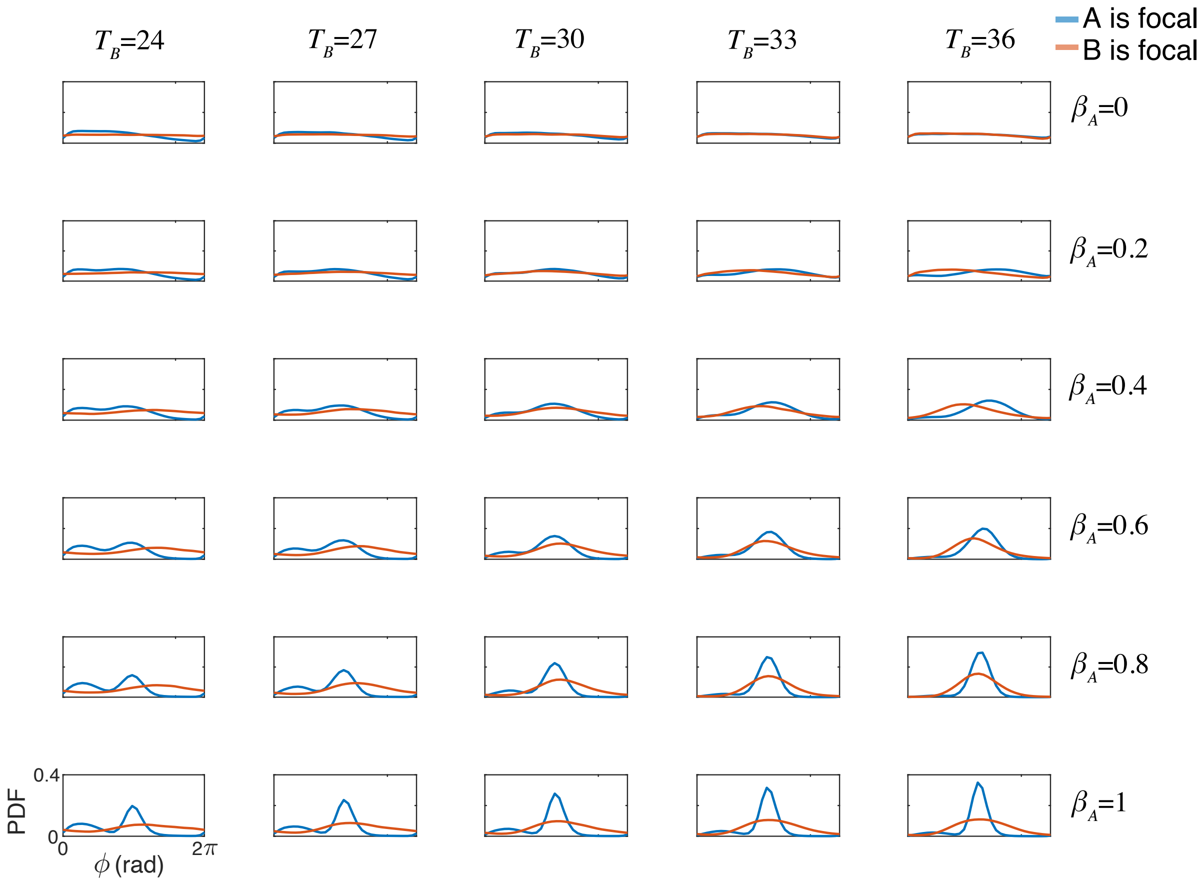

Supplementary Fig. 8: The distribution of phase differences ϕ between the two oscillators in the unidirectional model. Each plot corresponds to a different setting of β_A (the reciprocal oscillator) and T_B (the metronome) in timesteps.

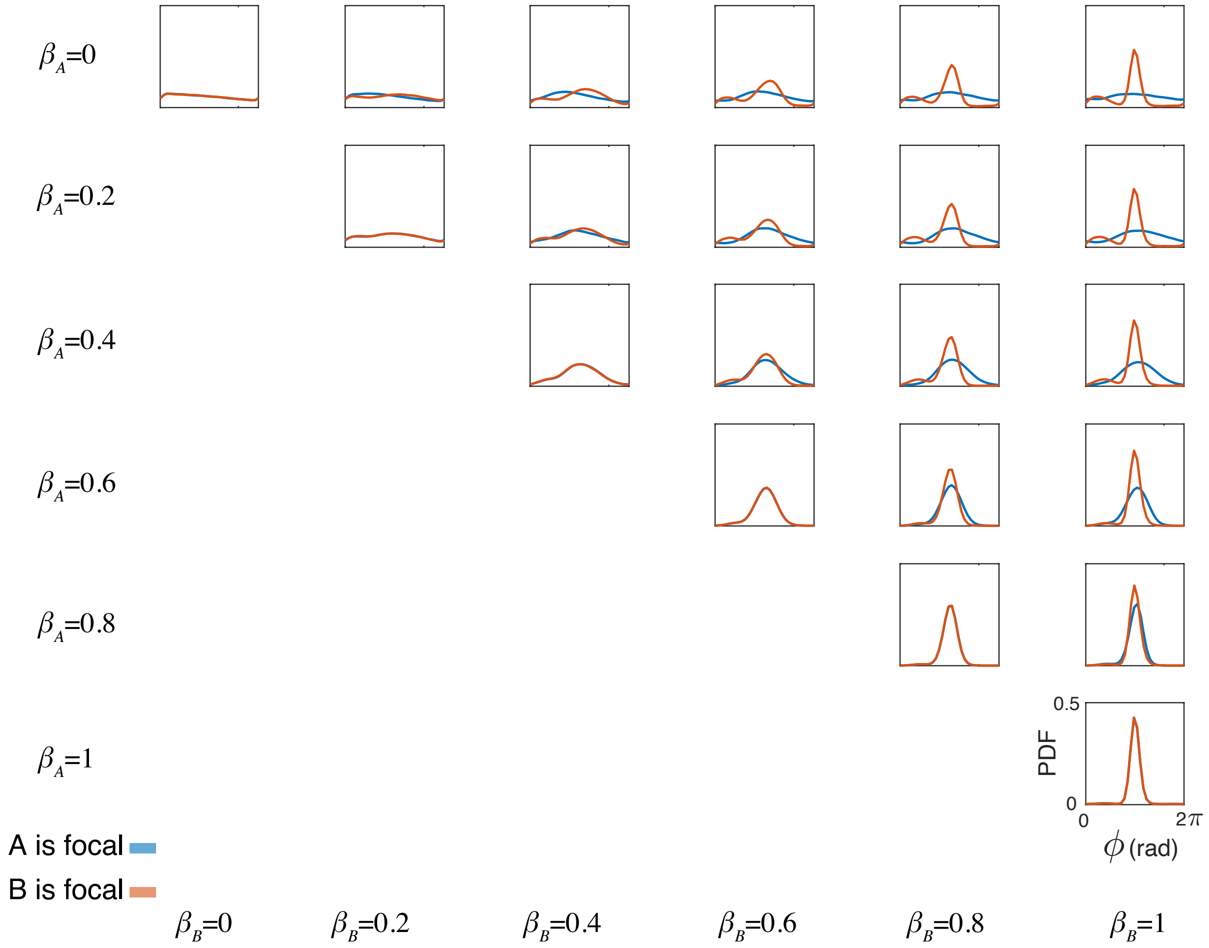

Supplementary Fig. 9: The distribution of phase differences ϕ between the two oscillators in the bidirectional model. Each plot corresponds to a different setting of β per each oscillator. Some plots are not shown due to symmetry.

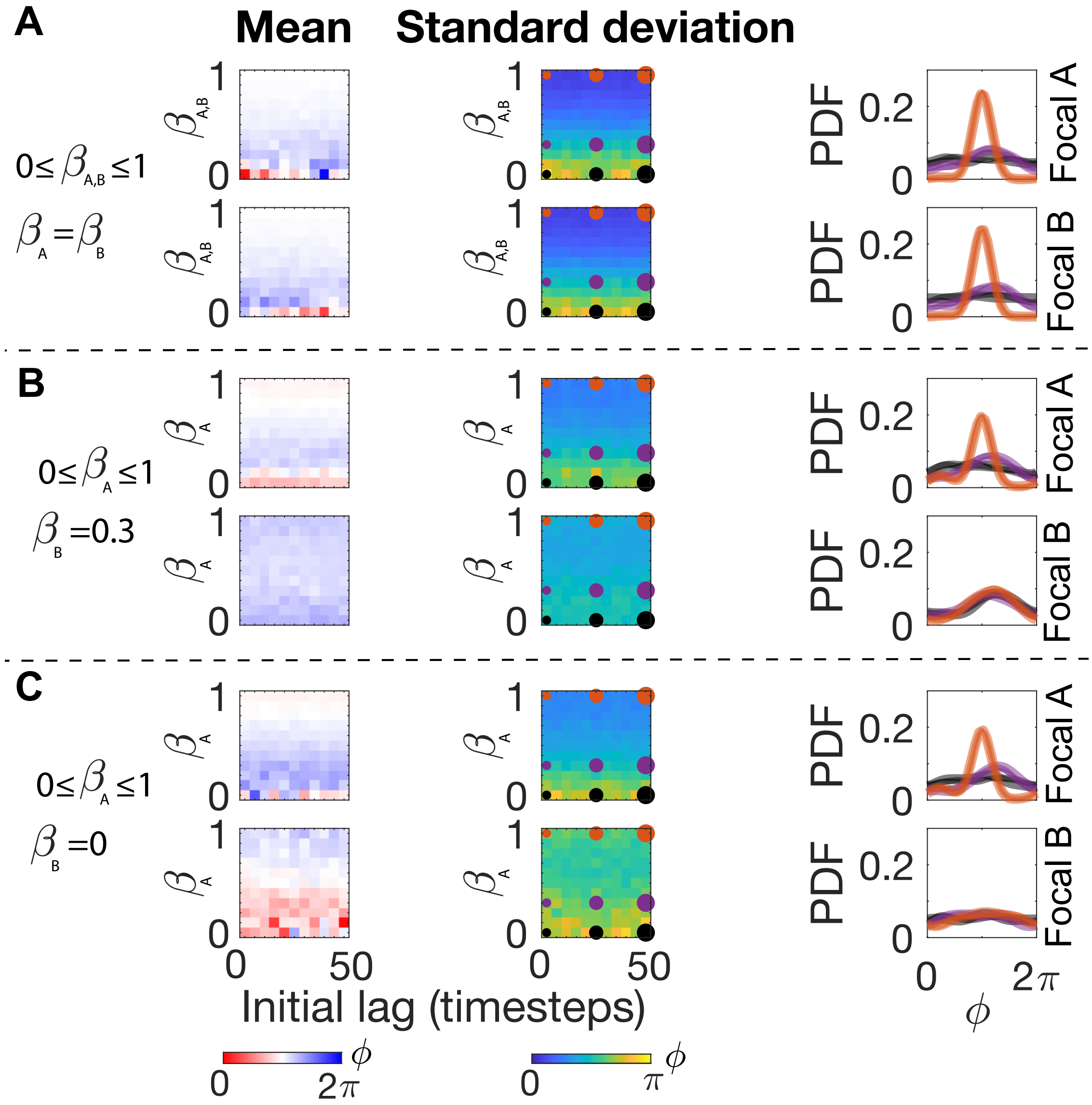

Supplementary Fig. 10: Parameter space exploration using different initial conditions to indicate stability. Each panel (A, B and C) consists of 6 subplots; in each, the top row shows results with respect to oscillator A as the focal, and bottom rows for B as the focal. ... (*caption continues on the next page*)

Supplementary Fig. 10: (*caption continued from the previous page*) ... (A) a model where both oscillators are interactive, and we explore the effect of different (but always equal) β values. The other parameter we varied here is the initial lag between them (on the x axis of the left and middle plots). (B) equivalent to (A), only now one of the oscillators is fixed at $\beta = 0.3$ and the other has different β values as shown on the y axis. (C) equivalent to (B), only now one of the oscillators is fixed at $\beta = 0$. The left and middle columns show circular mean and standard deviations respectively, obtained from the last recorded phase relationships from all the model realizations combined. The relative phase (ϕ, in a range from 0 to 2π) calculated from the timing of the neighbor's beat which occurred within the last full burst-and-glide period (from beat to beat) of the focal individual. Left side plots: Colorcode indicates the mean of the relative phase (ϕ) from red (0) through white (out-of-phase at π) to blue (at 2π). Middle plots: standard deviation of the relative phase (ϕ) colorcoded from blue through green to yellow. Right side plots: show full distributions from specific points in the parameter space depicted as points in the middle column. Line thickness and color correspond to the equivalent points.

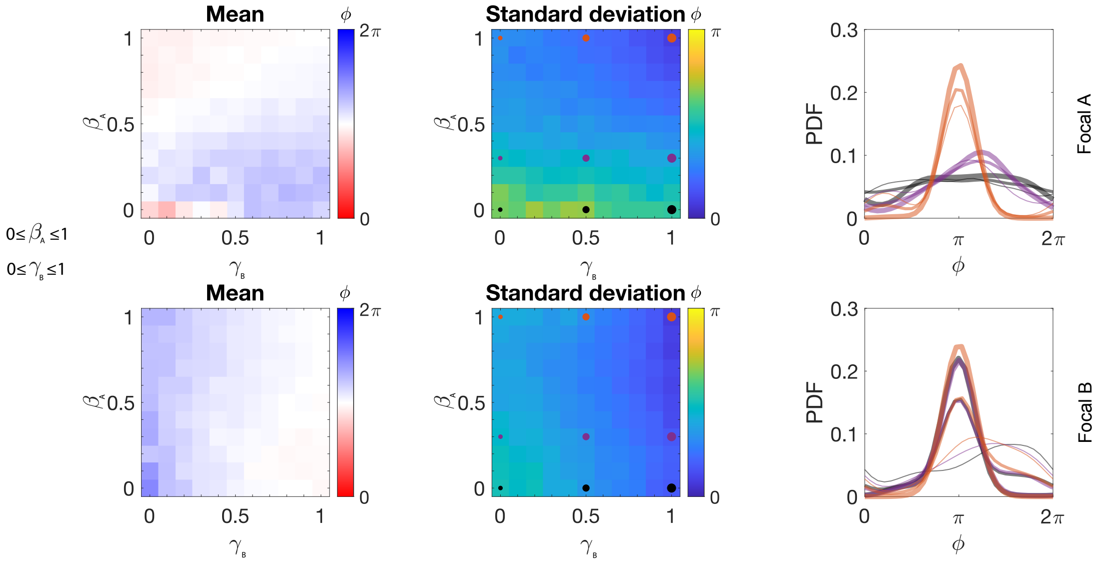

Supplementary Fig. 11: Exploring the transition from one- to two-way interactions. We vary γ, a parameter that controls the interactivity of one of the oscillators (here, oscillator B)—from acting as a metronome (fixed to $T_B = 30$ in simulation steps) to employing the PRC. For oscillator A we simply vary β. Top row shows results with respect to A as the focal, whereas bottom row is for B as the focal. The left and middle columns show circular mean and standard deviations respectively, obtained from the last recorded phase relationships from all the model realizations combined. The right side column shows full distributions from specific points in the parameter space depicted as points in the middle column. Line thickness and color corresponds to the equivalent points.

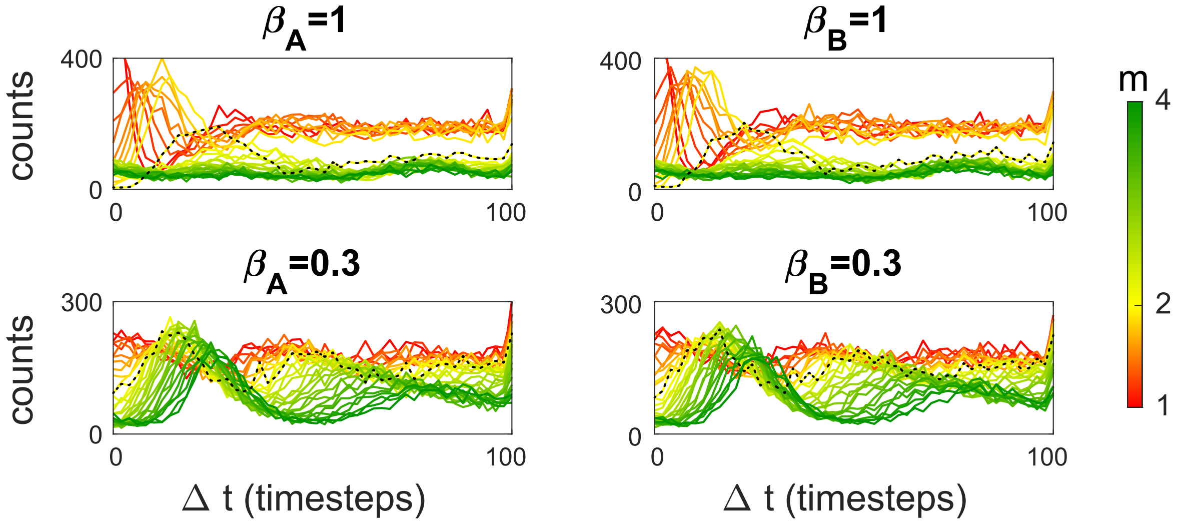

Supplementary Fig. 12: Effect of varying the slope of the PRC (m) on the time difference (Δt). All plots show distributions of the time differences between the two oscillators (left ones paired with the right ones). The color of the curves depicts the slope m in the function $y = mx$. We vary the values from 1 (the response y being identical to the input x; shown with red) up to 4 (shown with green) including 2 which is the value used in our model (shown with yellow and highlighted with a dashed line). Top plots show results for when both oscillators were set to $\beta = 1$, and bottom plots show results for coupling between oscillators with $\beta = 0.3$.

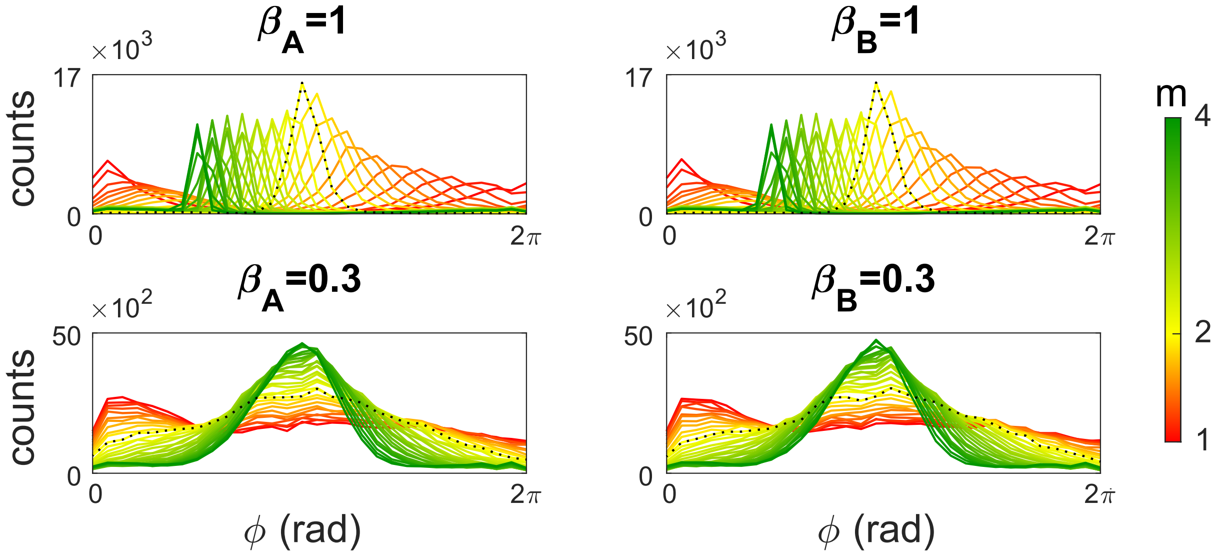

Supplementary Fig. 13: Effect of varying the slope of the PRC (m) on the phase difference (ϕ). All plots show distributions of the phase differences between the two oscillators. Other details are identical to the previous figure.

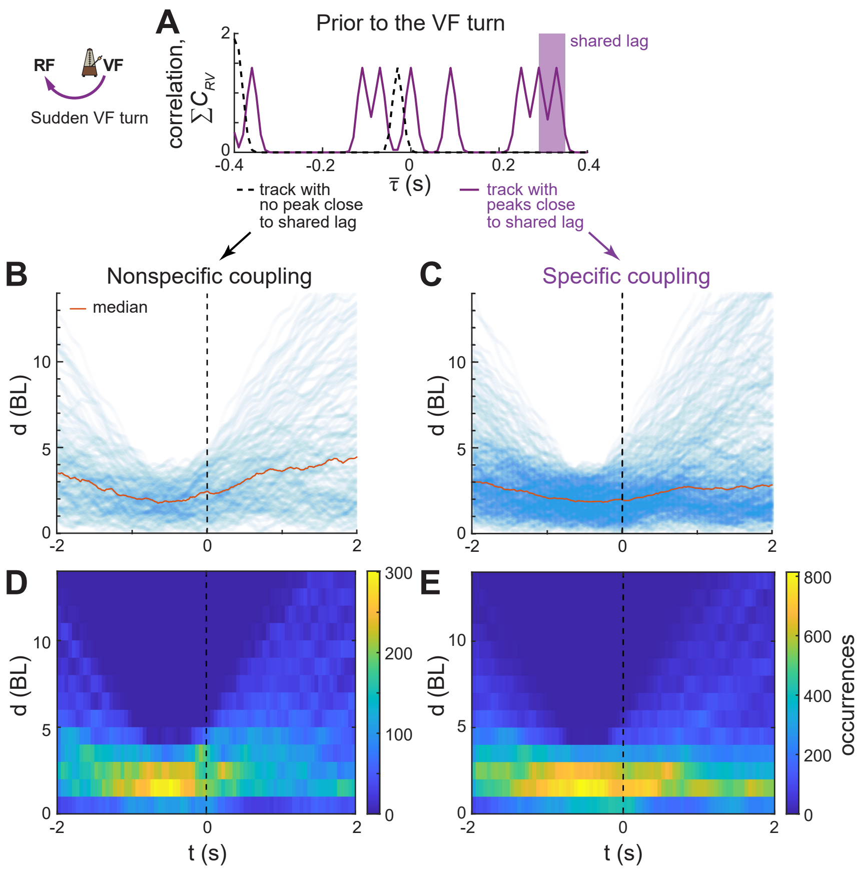

Supplementary Fig. 14: Experiment with the VF turns. (A) An explanation of how we define specific and nonspecific temporal coupling. The shared lag refers to the lag found in the nonreciprocal VF experiment (Fig. 2D). (B and C) Distance between the RF and VF before and after the VF turn—raw tracks from all events, overlaid on top of each other. (D and E) The same data as in B and C, presented as a heatmap for better visualization of the density. B and D show cases when the temporal coupling prior to the VF turn was not specific (i.e., no peaks in C_{RV} found in proximity to the shared lag from the nonreciprocal VF experiment (Fig. 2D), whereas C and E are for cases where the specific temporal coupling occurred (there were peaks in proximity to the shared lag).

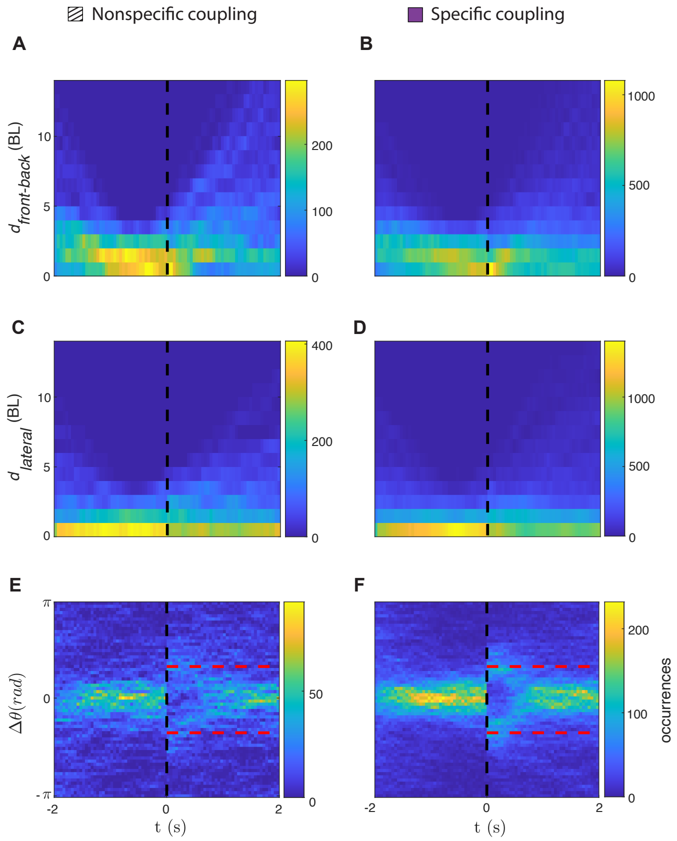

Supplementary Fig. 15: Spatial relationship between the RF and VF before and after the VF turn. Front-back distance between the fish over time, for nonspecific coupling (A), and specific coupling (B) (explained in S14). Lateral distance between the fish over time, for nonspecific coupling (C), and specific coupling (D). Relative orientation (degree of alignment) between the fish over time, for nonspecific coupling (E), and specific coupling (F).

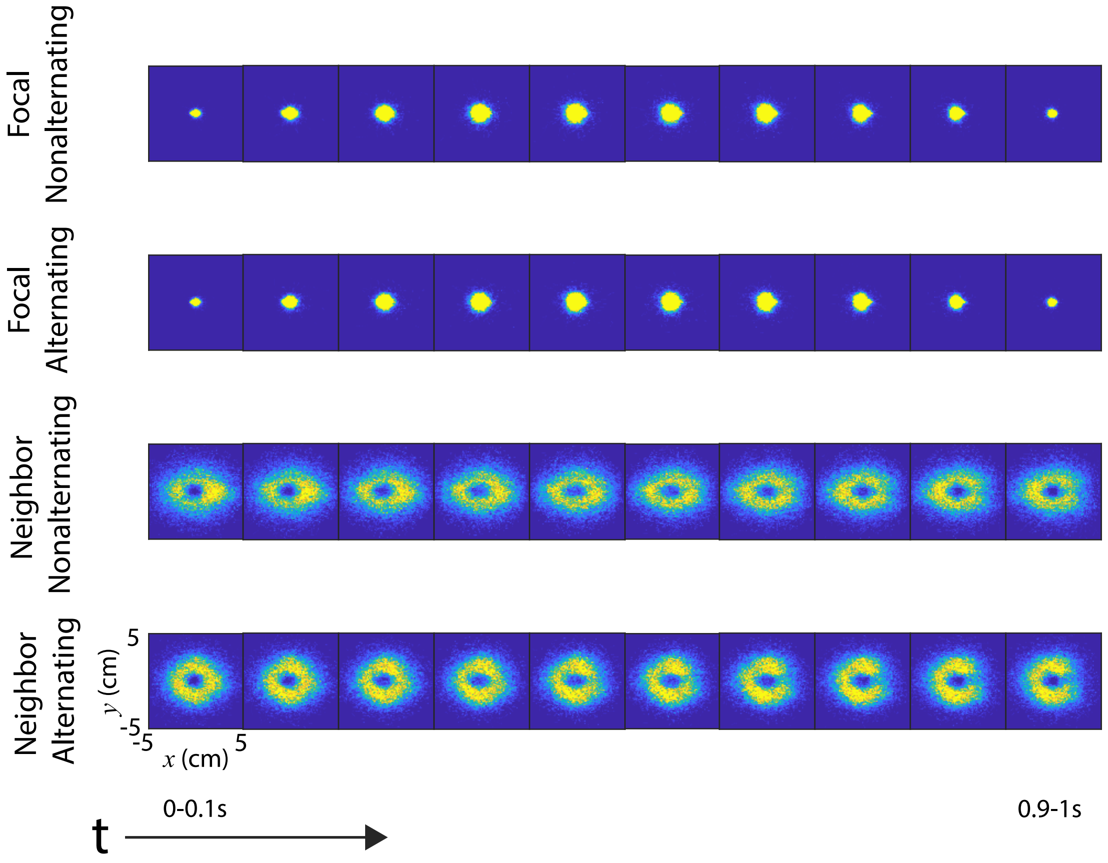

Supplementary Fig. 16: Explanation of the co-moving reference frame analysis. We obtain heatmaps for each time bin (of 0.1s) for the focal fish (top two rows) and the neighbor (bottom two rows). Each heatmap is ±5 cm in both axes around the reference fish.
