# Supplementary material for: Revealing the mechanism and function underlying pairwise temporal coupling in collective motion
Source: Nat Commun. 2024 May 22;15:4356. doi: 10.1038/s41467-024-48458-z (PMC11111445; doi:10.1038/s41467-024-48458-z)
Supplement: Supplementary file 3 — Reporting Summary [file 41467_2024_48458_MOESM3_ESM.pdf]

nature portfolio

Corresponding author(s): Guy Amichay, Mate Nagy, Iain D. Couzin

Last updated by author(s): Apr 22, 2024

Reporting Summary

Nature Portfolio wishes to improve the reproducibility of the work that we publish. This form provides structure for consistency and transparency in reporting. For further information on Nature Portfolio policies, see our Editorial Policies and the Editorial Policy Checklist.

Statistics

For all statistical analyses, confirm that the following items are present in the figure legend, table legend, main text, or Methods section.

n/a	Confirmed	
☐	☒	The exact sample size (*n*) for each experimental group/condition, given as a discrete number and unit of measurement
☐	☒	A statement on whether measurements were taken from distinct samples or whether the same sample was measured repeatedly
☐	☒	The statistical test(s) used AND whether they are one- or two-sided<br>*Only common tests should be described solely by name; describe more complex techniques in the Methods section.*
☒	☐	A description of all covariates tested
☒	☐	A description of any assumptions or corrections, such as tests of normality and adjustment for multiple comparisons
☐	☒	A full description of the statistical parameters including central tendency (e.g. means) or other basic estimates (e.g. regression coefficient) AND variation (e.g. standard deviation) or associated estimates of uncertainty (e.g. confidence intervals)
☐	☒	For null hypothesis testing, the test statistic (e.g. *F*, *t*, *r*) with confidence intervals, effect sizes, degrees of freedom and *P* value noted<br>*Give P values as exact values whenever suitable.*
☒	☐	For Bayesian analysis, information on the choice of priors and Markov chain Monte Carlo settings
☒	☐	For hierarchical and complex designs, identification of the appropriate level for tests and full reporting of outcomes
☒	☐	Estimates of effect sizes (e.g. Cohen's *d*, Pearson's *r*), indicating how they were calculated

Our web collection on statistics for biologists contains articles on many of the points above.

Software and code

Policy information about availability of computer code

Data collection	Experiments were conducted in a VR setup produced by loopbio GmbH. One can refer to Stowers et al. (2017) for the details of the fishVR setup. In addition, fish tracking was done using the TRex software (Walter and Couzin, 2021).<br><br>Stowers, J. R. et al. Virtual reality for freely moving animals. Nature Methods 14, 995–1002 (2017).<br><br>Walter, T. & Couzin, I. D. Trex, a fast multi-animal tracking system with markerless identification, and 2d estimation of posture and visual fields. Elife 10, e64000 (2021).
Data analysis	Data processing and analysis was done with MATLAB versions 2020a and 2023b. All codes that support the findings of this study are available on figshare with the identifier 10.6084/m9.figshare.25404004.

For manuscripts utilizing custom algorithms or software that are central to the research but not yet described in published literature, software must be made available to editors and reviewers. We strongly encourage code deposition in a community repository (e.g. GitHub). See the Nature Portfolio guidelines for submitting code & software for further information.

Data

Policy information about availability of data

All manuscripts must include a data availability statement. This statement should provide the following information, where applicable:

- Accession codes, unique identifiers, or web links for publicly available datasets
- A description of any restrictions on data availability
- For clinical datasets or third party data, please ensure that the statement adheres to our policy

The data that support the findings of this study are available in figshare with the identifier https://doi.org/10.6084/m9.figshare.c.7123501.v1

Research involving human participants, their data, or biological material

Policy information about studies with human participants or human data. See also policy information about sex, gender (identity/presentation), and sexual orientation and race, ethnicity and racism.

Reporting on sex and gender	N/A
Reporting on race, ethnicity, or other socially relevant groupings	N/A
Population characteristics	N/A
Recruitment	N/A
Ethics oversight	N/A

Note that full information on the approval of the study protocol must also be provided in the manuscript.

Field-specific reporting

Please select the one below that is the best fit for your research. If you are not sure, read the appropriate sections before making your selection.

☐ Life sciences ☒ Behavioural & social sciences ☐ Ecological, evolutionary & environmental sciences

For a reference copy of the document with all sections, see nature.com/documents/nr-reporting-summary-flat.pdf

Behavioural & social sciences study design

All studies must disclose on these points even when the disclosure is negative.

Study description	Quantitative experimental; including: lab-based measurements of behavior, controlled experiments in VR, computational modeling and analyses of thereof.
Research sample	276 zebrafish of age 24 to 26 days post fertilization raised in a room at 28 degrees on a 12-h light, 12-h dark cycle. The sample is representative--we reach highly significant results and this is the range that was employed in previous works on social interactions of this species.
Sampling strategy	We determined sample size according to the three Rs principle (Replacement, Reduction and Refinement). We used all data without sub-sampling, i.e., this was random (fish were chosen at random).
Data collection	All experiments were conducted on 1 cm ± 0.1 cm long zebrafish of age 24 to 26 days post-fertilization raised in a room at 28 °C on a 12 h light, 12 h dark cycle (light switching on and off and 7 am and 7 pm). The fish were bred and raised by the animal care staff of the Department of Collective Behaviour, Max Planck Institute of Animal Behaviour in an animal facility at the University of Konstanz. Fish were transferred to the experimental room at least 12 h prior to the experiments in water from their holding tanks. This ensured that the water quality in the experimental room was the same as in their holding facility. This water was also used in the experimental setups (either the arenas for 2 real fish experiments or the fishVR setup for virtual reality experiments) where water changing was done once a day. All the fish were na¨ıve, and chosen at random from their holding tanks. All experiments were conducted in accordance with the animal ethics permit approved by Regierungsprasidium Freiburg, G-17/170, G-17/46 and G-21/153.<br><br>The majority of the experiments were executed by technicians in the lab, that were blinded to experimental conditions and / or the study hypothesis.
Timing	Pairs of real fish were tracked in two separate times: the first between 6.12.2017-22.01.2018 and the second batch between 7-14.09.2020.<br>Nonreciprocal virtual fish experiments were conducted between 23-30.5.2020.

	Reciprocal virtual fish experiments were conducted between 05.02.2024-13.03.2024.
Data exclusions	To account for errors in detection from the tracking algorithm of the pairs, we omitted from our analysis all data with undefined x, y or speed values. In addition, we removed minima of speed (the initiation of the bursts) that were above 5 cm/s or that weren't followed by a pronounced acceleration (100 ms after the minima the increase in speed was <2 cm/s) to only account for actual bursts and not small variations in speed.<br><br>Specifically for VR experiments, we omitted cases where we detected errors in the VR output, whereby the speed of the VF wasn't according to what we had assigned. That is, if we detected too low or too high speeds (< 0.001m/s or > 0.11m/s) for more than 5 frames within our window of analysis (100 frames in the case of the nonreciprocal VF with no VF turns and 300 frames in the case with the VF turns). In the case of the reciprocal VF, where the speed profile isn't fully determined prior to the experiment, we omit cases according to extreme values of VF acceleration (numerical differentiation of the speed)—that is, if we had more than 5 frames of > 0.05m/s^2 or < −0.05m/s^2 in a 100 frame window.<br><br>This description is given in the manuscript as well.
Non-participation	No participants were involved in the study.
Randomization	As each fish was used only once, there was no need to randomize them.

Reporting for specific materials, systems and methods

We require information from authors about some types of materials, experimental systems and methods used in many studies. Here, indicate whether each material, system or method listed is relevant to your study. If you are not sure if a list item applies to your research, read the appropriate section before selecting a response.

Materials & experimental systems

n/a	Involved in the study	
☒	☐	Antibodies
☒	☐	Eukaryotic cell lines
☒	☐	Palaeontology and archaeology
☐	☒	Animals and other organisms
☒	☐	Clinical data
☒	☐	Dual use research of concern
☒	☐	Plants

Methods

n/a	Involved in the study	
☒	☐	ChIP-seq
☒	☐	Flow cytometry
☒	☐	MRI-based neuroimaging

Animals and other research organisms

Policy information about studies involving animals; ARRIVE guidelines recommended for reporting animal research, and Sex and Gender in Research

Laboratory animals	Zebrafish (Danio rerio), Konstanz wild type, age 24-26 days post-fertilization
Wild animals	N/A
Reporting on sex	Our study deals with juveniles, where sexual differences aren't likely to yet play a prominent role. We randomly chose fish from a holding tank including both sexes, and we used them in the study without distinguishing their sex.
Field-collected samples	did not involve
Ethics oversight	All experiments were conducted in accordance with the animal ethics permit approved by Regierungsprasidium Freiburg, G-17/170, G-17/46 and G-21/153.

Note that full information on the approval of the study protocol must also be provided in the manuscript.

Plants

Seed stocks

Report on the source of all seed stocks or other plant material used. If applicable, state the seed stock centre and catalogue number. If plant specimens were collected from the field, describe the collection location, date and sampling procedures.

Novel plant genotypes

Describe the methods by which all novel plant genotypes were produced. This includes those generated by transgenic approaches, gene editing, chemical/radiation-based mutagenesis and hybridization. For transgenic lines, describe the transformation method, the number of independent lines analyzed and the generation upon which experiments were performed. For gene-edited lines, describe the editor used, the endogenous sequence targeted for editing, the targeting guide RNA sequence (if applicable) and how the editor was applied.

Authentication

Describe any authentication procedures for each seed stock used or novel genotype generated. Describe any experiments used to assess the effect of a mutation and, where applicable, how potential secondary effects (e.g. second site T-DNA insertions, mosiacism, off-target gene editing) were examined.
